# Prenatal Organochlorine Exposure and Measures of Behavior in Infancy Using the Neonatal Behavioral Assessment Scale (NBAS)

**DOI:** 10.1289/ehp.10553

**Published:** 2008-01-24

**Authors:** Sharon K. Sagiv, J. Kevin Nugent, T. Berry Brazelton, Anna L. Choi, Paige E. Tolbert, Larisa M. Altshul, Susan A. Korrick

**Affiliations:** 1 Department of Environmental Health, Harvard School of Public Health, Boston, Massachusetts, USA; 2 Brazelton Institute, Division of Child Development, Children’s Hospital, Harvard Medical School, Boston, Massachusetts, USA; 3 University of Massachusetts at Amherst, Amherst, Massachusetts, USA; 4 Department of Environmental and Occupational Health, Rollins School of Public Health, Emory University, Atlanta, Georgia, USA; 5 Channing Laboratory, Department of Medicine, Brigham and Women’s Hospital, Harvard Medical School, Boston, Massachusetts, USA

**Keywords:** behavior, infant, organochlorines, *p,p′*-dichlorodiphenyl dichloroethene (DDE), poly-chlorinated biphenyls (PCBs)

## Abstract

**Background:**

Previous literature suggests an association between organochlorines and behavioral measures in childhood, including inattention.

**Objective:**

This study was designed to assess whether prenatal organochlorine exposure is associated with measures of attention in early infancy.

**Methods:**

We investigated an association between cord serum polychlorinated biphenyls (PCBs) and *p,p*′-dichlorodiphenyl dichloroethene (DDE) levels and measures of attention from the Neonatal Behavioral Assessment Scale (NBAS) in a cohort of 788 infants born 1993–1998 to mothers residing near a PCB-contaminated harbor and Superfund site in New Bedford, Massachusetts.

**Results:**

Medians (ranges) for the sum of four prevalent PCB congeners and DDE levels were 0.19 (0.01–4.41) and 0.30 (0–10.29) ng/g serum, respectively. For the 542 subjects with an NBAS exam at 2 weeks, we observed consistent inverse associations between cord serum PCB and DDE levels and NBAS measures of alertness, quality of alert responsiveness, cost of attention, and other potential attention-associated measures including self-quieting and motor maturity. For example, the decrement in quality of alert responsiveness score was −0.51 (95% confidence interval, −0.99 to −0.03) for the highest quartile of exposure to the sum of four prevalent PCB congeners compared with the lowest quartile. We found little evidence for an association with infant orientation, habituation, and regulation of state, assessed as summary cluster measures.

**Conclusions:**

Our findings provide evidence for an association between low-level prenatal PCB and DDE exposures and poor attention in early infancy. Further analyses will focus on whether organochlorine-associated decrements in attention and attention-related skills in infancy persist in later childhood.

Organochlorines, including polychlorinated biphenyls (PCBs) and *p,p*′-dichlorodiphenyl dichloroethene (*p,p*′-DDE), the major degradation product of *p,p*′-dichlorodiphenyl trichloroethane (*p,p*′-DDT), though banned in the U.S. in the 1970s, are highly persistent in the environment and in human tissue. These contaminants cross the placenta and have been associated with reduced birth size (Fein et al. 1984; Hertz-Picciotto et al. 2005; Longnecker et al. 2001; Sagiv et al. 2007) and effects on subsequent neurodevelopment (Schantz et al. 2003).

Several previous studies have investigated the association between prenatal PCB exposures and behavior in infancy using the Neonatal Behavioral Assessment Scale (NBAS) (Jacobson et al. 1984a; Lonky et al. 1996; Rogan et al. 1986; Stewart et al. 2000). These studies, which summarize the NBAS scores using seven summary cluster measures developed by Lester et al. (1982) and later revised by Jacobson et al. (1984b), found the most consistent associations with increased number of abnormal reflexes (particularly hyporeflexia); in addition, decreased autonomic maturity and habituation, and, less commonly, poorer range of state scores were found in association with PCB exposures. Studies have also found associations between PCBs and infant visual recognition memory, potentially an early indicator of attention, using the Fagan Test of Infant Intelligence (Darvill et al. 2000; Jacobson et al. 1985).

Early life experience (including prenatal exposures) and health status are increasingly recognized as having enormous potential to affect subsequent childhood and, even adult, health (Kuh et al. 2003). However, behavioral or related functional health outcomes are difficult to measure in early infancy; there are few tests available for this age group, and generally such assessments have limited reproducibility and poor long-term predictive value (Gorski et al. 1987). Although an efficient data reduction technique, the seven summary NBAS clusters may not detect important behavior patterns captured by individual NBAS items.

For the current study, we hypothesized a specific association between prenatal organo-chlorine exposure and measures of attention in early infancy, and used individual NBAS items to characterize young infants’ attention skills. Previous literature reports associations between PCBs and attention among school-age children and adults (Grandjean et al. 2001; Jacobson and Jacobson 1996, 2003; Peper et al. 2005; Vreugdenhil et al. 2004). To investigate whether these associations could be detected in early infancy, we conducted a prospective cohort study of cord serum organochlorines and attention-related outcomes as measured by the NBAS among infants born to mothers residing near a PCB-contaminated harbor.

## Materials and Methods

### Study population

Study participants were a subset of infants from a birth cohort whose mothers resided adjacent to a PCB-contaminated harbor in New Bedford, Massachusetts, and were recruited at the time of the infant’s birth between 1993 and 1998. The parent cohort includes 788 mother–infant pairs participating in ongoing studies of prenatal PCB and organochlorine pesticide exposure and subsequent infant and child development. The cohort was recruited from a local hospital with approximately 2,000 births per year, about 10% of whom were available for recruitment during times when a study examiner was on site, and met study eligibility criteria. Mother–infant pairs were eligible to participate if the mother was ≥ 18 years of age, had lived in one of four towns (New Bedford, Acushnet, Fairhaven, Dartmouth) adjacent to the contaminated New Bedford Harbor for at least the duration of her pregnancy, and spoke English or Portuguese. Infants too ill to undergo neonatal examination or born by cesarean section were excluded. Preterm infants (< 37 weeks gestation) and multiple births were excluded from the current analysis.

### Laboratory measurements of exposure

Cord blood samples for organochlorine analyses were collected at the infant’s birth; the serum fraction was removed after centrifugation and stored at −20°C. All sample analyses were performed by the Harvard School of Public Health Organic Chemistry Laboratory (Boston, Massachusetts). Laboratory personnel were blinded to infant outcomes. Cord serum samples were analyzed for 51 individual PCB congeners and two chlorinated pesticides [*p,p*′-DDE and hexachlorobenzene (HCB)]. Laboratory analytic methods and quality control procedures are described elsewhere (Korrick et al. 2000). Briefly, liquid–liquid extraction was used according to procedures developed by the Centers for Disease Control and Prevention with modifications to conform to ultratrace-level analyses (Korrick et al. 2000). Extracts were analyzed by gas chromatography with electron capture detection on a Hewlett-Packard 5890 Series II GC (Hewlett Packard, Palo Alto, CA) with temperature and pressure programming capabilities and a split/splitless injector. Samples with possible phthalate contamination or a coeluting substance detected in blanks were checked by confirmatory analyses on a Hewlett-Packard 6890 GC with a Micro-ECD and a capillary column of different polarity. Where results differed, the lower value—considered more accurate because it indicated separation of the PCB congener from an interfering coeluting peak—was reported (Erickson 1997).

Quantitation was based on the response factor of each individual PCB congener or pesticide relative to an internal standard. PCB concentrations were reported as individual congeners, after the amount of analyte in the procedural blank was subtracted, and as the sum of all congeners assayed (∑PCB) in units of nanograms per gram serum. Lipid content could not be determined for study subjects because of insufficient sample volume (1.5–4 mL) and was therefore measured for 12 randomly selected cord bloods from discarded, anonymous samples collected at the study recruitment site; values were reproducible (1.7 ± 0.3 g/L) and consistent with lipid content in cord blood reported elsewhere (1.8 ± 0.07 g/L) (Denkins et al. 2000).

The method detection limits (MDLs) for individual PCBs ranged from 0.002 to 0.04 ng/g of serum, with most MDLs < 0.01 ng/g; respective MDLs for DDE and HCB in serum were 0.07 and 0.02 ng/g (Korrick et al. 2000). Ninety-six percent of samples had DDE levels above the MDL; from < 1% (congener 22) to 91% (congener 153) of samples had PCB congeners above the MDL (Korrick et al. 2000). Where no measurable quantity of analyte was detected, a value of zero was used in our analyses. We used quantifiable values below the detection limit to optimize statistical power and avoid biased exposure estimates associated with censoring at the MDL (Kim et al. 1995). Reproducibility of serum analyses was good; the ∑PCB within-batch coefficient of variation (CV) was 3% and the between-batch CV was 20% over the 5 years of analysis, with similar performance for pesticides.

Cord blood samples for lead measurement were analyzed at the Harvard School of Public Health Trace Metals Analysis Laboratory (Boston, Massachusetts). Samples were collected at birth in EDTA-containing Vacutainer tubes for trace metal analyses. Analyses employed isotope dilution inductively coupled plasma mass spectrometry (Sciex Elan 5000; PerkinElmer, Norwalk, CT) with standard instrument operating and data collection parameters. Quality control and assurance procedures included analyses of procedural blanks, duplicates, spiked samples, and standard reference material to monitor for contamination, accuracy, and recovery rates. Recovery rates for QC and spiked samples were 90–110%, and precision was < 5%. The detection limit was 0.02 μg/dL.

### Neonatal outcome assessment

Newborns were examined twice with the NBAS (Brazelton and Nugent 1995). The first assessment took place between the first and third days of life (referred to as the birth exam), and the second was administered between 5 and 22 days, 80% of which fell between 8 and 20 days, (referred to as the 2-week exam). The NBAS assesses the infant’s behavioral capacities, including his or her ability to respond to the environment, such as the ability to orient and habituate to visual or auditory stimuli, both animate and inanimate; the quality of motor tone and activity levels; and the infant’s level of state regulation (i.e., amount of crying and the infant’s capacity to regulate his or her asleep, alert, crying states). The NBAS exam takes approximately 30 min to administer and contains 28 behavioral items, 18 elicited items (including neonatal reflexes), and up to 9 supplementary items, designed to capture the quality of newborn behavioral responsiveness. Each of the NBAS behavioral items is assigned a score (ranging from 1 to 9), according to established scoring criteria, with a higher score typically indicating better performance and a lower score indicating poorer performance (there are some items in which the opposite is the case or where the midpoint is optimal). Neonatal assessments were performed by three study staff members trained in administration and scoring according to the inter-rater reliability criteria established by the NBAS manual (Brazelton and Nugent 1995). Interobserver scoring agreement was calculated before the beginning of the study and then at least biannually thereafter; inter-rater agreement was established at ≥ 90%.

The focus of the current study was infant attention, and we analyzed eight *a priori* selected NBAS behavioral items to identify the infant’s capacity for attention or abilities potentially associated with attention, such as state regulation and motor maturity. Table 1 lists all outcomes analyzed. The individual behavioral items analyzed for this study were alertness, consolability, self-quieting activity, hand-to-mouth facility, irritability, elicited and spontaneous activity, and motor maturity. NBAS supplementary items were designed to evaluate the infant’s ability to cope with the examination and maintain an alert state (Brazelton and Nugent 1995). Two supplementary items measuring alertness were selected for inclusion in the analysis: *a*) quality of alert responsiveness, which assesses the level of “processing” alertness as opposed to a simple awake “eyes open” state, and *b*) cost of attention, which measures the degree to which the motor and physiologic systems were stressed as a result of the infant’s efforts to attend to the stimuli.

As a secondary analysis we also analyzed three of six previously defined behavioral clusters (Jacobson et al. 1984b; Lester et al. 1982) selected *a priori* as those that potentially reflect skills associated with attention. Each cluster score was computed as the mean of individual behavioral items associated with that cluster; where more than half the items for a cluster were missing, the cluster score was omitted. The three clusters were *a*) orientation [tracking of visual animate (human face) and inanimate (red ball) stimuli and auditory animate (human voice) and inanimate (rattle) stimuli and the duration and quality of the infant’s alertness]; *b*) response decrement or habituation (habituation to light, rattle, bell, and pin prick stimuli); and *c*) regulation of state (self-quieting activity and hand-to-mouth facility).

Several subjects were missing scores for all individual items in the orientation cluster, presumably because they never achieved an alert or awake state in which they were able to focus attention on visual or auditory stimuli. Failure to reach a robust alert state for evaluation may be informative of attention-associated problems. To explore this possibility, we created a dichotomous variable called “never in state to do orientation items” and coded as *a*) missing data for all orientation items and *b*) data on at least one orientation item.

The main analyses focused on NBAS outcomes collected at the 2-week exam, which may be a better indicator of infant behavior because the infant will have likely recovered from the demands associated with the birth experience (Lester 1983; Lester et al. 1985). We also assessed whether the infant’s ability to recover from the birth was associated with organochlorine exposure by comparing performance for the individual items on the birth exam with the 2-week exam. For this analysis we focused on whether an infant scoring in the lowest two-thirds of the item on the birth exam advanced to a better score on the 2-week exam (i.e., lowest third advanced to middle or highest third, or middle third advanced to highest third). Infants scoring in highest third on the birth exam were omitted because they did not have the opportunity to improve their score. We created a dichotomous outcome and assessed whether exposure to PCBs or DDE was associated with failure to recover or advance to a higher score.

### Statistical analysis

We measured 51 PCB congeners and investigated behavioral outcomes in relation to two congener groups: *a*) the sum of four prevalent PCB congeners: 118, 138, 153, and 180; and *b*) the computed toxic equivalent (TEQ) for the sum of the five dioxin-like mono-*ortho* PCB congeners measured: 105, 118, 156, 167, and 189, computed on a lipid basis and weighted with toxic equivalency factors (TEFs) (Van den Berg et al. 1998). Although these PCB groupings are highly correlated with each other (correlation coefficients = 0.94), we conducted separate analyses to explore potentially different toxicologic mechanisms of action for dioxin-like versus non-dioxin-like congeners (Schantz et al. 2003). We also investigated associations with *p,p*′-DDE. A nonlinear effect of organo-chlorines was investigated by dividing exposure into quartiles of the distribution.

We used linear regression analysis to estimate differences in score by level of exposure for the NBAS outcomes scored on a continuous scale; a lower score was associated with a poorer outcome. We also dichotomized 3 outcome measures: *a*) irritability [high (score 7–9) vs. normal (score 4–6); only 13 subjects scored < 4]; *b*) missing orientation data (missing data on all orientation items vs. data on one or more item); and *c*) failure to recover from the birth experience (failure to advance to a better score from the birth exam to the 2-week exam). Risk ratios were generated for these dichotomous outcomes with log risk models.

Data on covariates came primarily from a questionnaire administered at the 2-week exam. Questions were asked about maternal medical and reproductive histories; typical diet; alcohol, tobacco, and illicit drug use; education; race and ethnicity; occupational and exposure histories potentially relevant to exposures or outcomes of interest; and household income. Trained study personnel also reviewed hospital medical records for study mothers and their infants, at which time details of the mother’s obstetric history, labor, and delivery and of the infant’s newborn physical exam were recorded.

Covariates included *a priori* in the model were infant’s age at the NBAS exam, time since last feeding, NBAS examiner and infant birth year. We also evaluated confounding by infant sex and cord blood lead level, and maternal age, race/ethnicity, education, birthplace, marital status, household income, parity, obstetric (OB) risk score, breast-feeding, average cigarette smoking and alcohol consumption during pregnancy, and illicit drug use in the year before birth and diet during pregnancy, including local fish consumption and overall fish consumption, regardless of source.

The OB risk score was a modified score derived from a scoring system created by Hobel et al. (1973) used to identify pregnancies that are at high risk. The original score includes the weighted sum of 126 items that incorporate prenatal, intrapartum, and neonatal risk factors. Our score used 33 items available for the study population and for which independent covariates were not in our models, including advanced maternal age, prepregnancy weight, parity, VDRL serology status; history of hypertension, cystitis, diabetes, thyroid disease, anemia; among prior births: preterm delivery, cesarean section, or congenital anomalies; for the current pregnancy: eclampsia, preeclampsia, pyelonephritis, viral disease, premature rupture of membranes, precipitous labor, prolonged labor, prolonged second-stage labor, medical induction of labor, administration of pitocin, forceps or suction/vacuum delivery, meconium staining, prolapsed cord, and fetal bradycardia; and for the current infant: Apgar at 1 and 5 min, whether resuscitation was required, congenital anomalies, cardiac anomalies, respiratory abnormalities, and jaundice. Use of the summary OB risk score requires far fewer parameters in the multivariable model and allowed for more precise effect estimation.

We included covariates that improved the fit of the model, demonstrated by a statistically significant partial *F*-test (α < 0.10) and investigated whether inclusion of these variables in the model had a meaningful impact on the exposure effect estimate. Individual NBAS items fell into three broad domains: *a*) measures of attention (alertness, quality of alert responsiveness and cost of attention), *b*) measures of state (consolability, self-quieting activity, hand-to-mouth facility, irritability and never in state to do orientation items), and *c*) measures of motor function (elicited and spontaneous activity, and motor maturity). For consistency, the set of covariates included within each domain were kept constant. Estimates of effect and 95% confidence intervals across quartiles of exposure were generated with multivariable models. We computed *p*-for-trend to assess monotonic linear trends in the data using the median exposure value for each quartile.

The study protocol was reviewed and approved by the human subjects committees of Harvard School of Public Health and Brigham and Women’s Hospital in Boston and of St. Luke’s Hospital, the New Bedford site of Southcoast Hospitals Group, New Bedford. Written informed consent was obtained from all participating families before study evaluation.

## Results

Among the 788 infants included in the initial study population, we excluded 6 twins, 24 singleton preterm births, and 36 infants without serum organochlorine measures. Of the remaining 722 infants, 539 had measures for the birth NBAS assessment, 542 for the 2-week assessment, and 408 infants were administered both exams. Table 2 shows characteristics for the 542 mother–infant pairs with the 2-week exam. Mothers were mostly white, > 80% graduated from high school, more than a third were low income (household income < $20,000 per year), a little more than half were married, and almost a third smoked during their pregnancy. The mean gestational age at birth and mean birth weight were 39.8 weeks and 3,401 g, respectively, and 37% of infants were breast-fed for at least 1 month. The median cord serum level for subjects included in this analysis was 0.19 ng/g (range, 0.01–4.41 ng/g serum) for the sum of four prevalent PCB congeners and 0.30 ng/g (range, 0–10.27 ng/g serum) for DDE.

**Figure 1:**
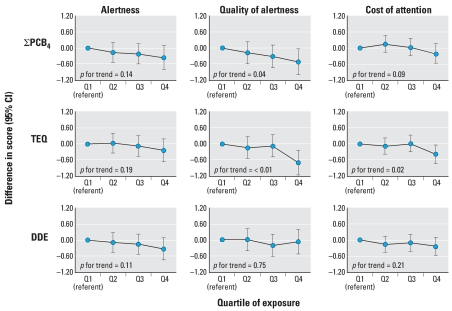
Associations and 95% confidence intervals (CIs) between cord serum levels of the sum of four PCB congeners (118, 138, 153, 180), the sum of mono-*ortho* TEF-weighted PCB congeners (TEQ), and DDE, and 2-week NBAS measures of attention (alertness, quality of alert responsiveness, and cost of attention), adjusted for infant’s age at exam, birth year, time since last feeding, NBAS examiner, and maternal age, education, marital status, parity, smoking during pregnancy, OB risk score, and cord blood lead level for term infants born in New Bedford, 1993–1998.

Figure 1 shows inverse associations for the two PCB congener groups as well as DDE and measures of attention, including alertness, quality of alert responsiveness, and cost of attention. Though the *p*-for-trend was statistically significant for just one-third of these associations, most of the graphs show a consistent decline in attention-related scores with increasing quartile of exposure.

**Figure 2:**
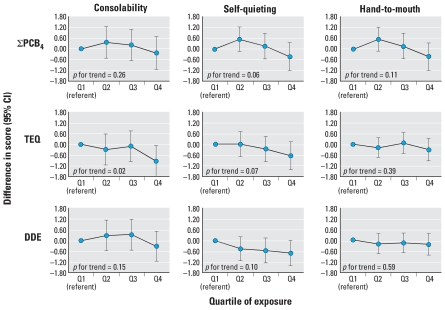
Associations and 95% confidence intervals (CIs) between cord serum levels of the sum of four PCB congeners (118, 138, 153, 180), the sum of mono-*ortho* TEF-weighted PCB congeners (TEQ), and DDE, and 2-week NBAS continuous measures of state (consolability, self-quieting, and hand-to-mouth facility), adjusted for infant’s age at exam, birth year, time since last feeding, NBAS examiner, and maternal age, education, breast-feeding, household income, and OB risk score for term infants born in New Bedford, 1993–1998.

**Figure 3:**
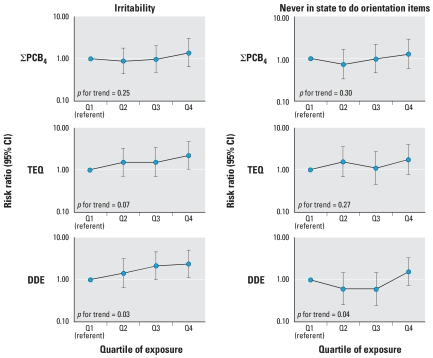
Associations and 95% confidence intervals (CIs) between cord serum levels of the sum of four PCB congeners (118, 138, 153, 180), the sum of mono-*ortho* TEF-weighted PCB congeners (TEQ), and DDE, and 2-week NBAS dichotomous measures of state [irritability (high score 7–9 vs. normal score 4–6) and never in state to do orientation items (all orientation items missing vs. data on at least one orientation item)], adjusted for infant’s age at exam, birth year, time since last feeding, NBAS examiner, and maternal age, education, breast-feeding, household income, and OB risk score for term infants born in New Bedford, 1993–1998.

There were less consistent associations between cord serum organochlorines and state-related items, including consolability, self-quieting activity, and hand-to-mouth facility (Figure 2). We observed inverse associations between only a few organochlorines and state-associated outcomes, and predominantly for self-quieting activity. Irritability was associated with the sum of mono-*ortho* TEF-weighted PCB congeners (TEQ) and DDE (Figure 3), as shown by increasing risk ratios for high versus normal irritability across quartiles of cord serum levels and significant or near significant *p*-values for trend. There was less evidence for increased risk of never being in the appropriate state to respond to orientation (missing data for all orientation items) across quartiles of cord serum PCBs, TEQ, and DDE.

**Figure 4:**
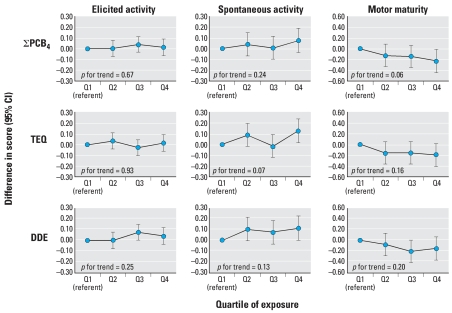
Associations and 95% confidence intervals (CIs) between cord serum levels of the sum of four PCB congeners (118, 138, 153, 180), the sum of mono-*ortho* TEF-weighted PCB congeners (TEQ), and DDE, and 2-week NBAS measures of motor function (elicited activity, spontaneous activity, and motor maturity), adjusted for infant sex, age at exam, birth year, time since last feeding, NBAS examiner, and maternal birthplace, breast-feeding, and OB risk score for term infants born in New Bedford, 1993–1998.

Less consistent associations were also found for motor outcomes, though motor maturity did appear to decline with increasing cord serum PCB, TEQ, and DDE levels (Figure 4). There appeared to be a positive association between cord serum organochlorines and spontaneous activity. There were no consistent associations between organochlorines and any of the three cluster measures (orientation, habituation, and regulation of state; data not shown).

The general pattern of these findings was unchanged when a narrower window of time (10–17 days after birth) was used for the 2-week exam. Findings also remained unchanged when more recent TEF weights were applied (Van den Berg et al. 2006).

Subjects had to have data for both the birth and the 2-week exams to be included in the failure-to-recover analysis. Depending on the item, between 59 and 358 subjects were included. There were no consistent patterns found for PCBs, TEQ, or DDE and failure to recover for any of the NBAS outcomes.

## Discussion

Our approach to analyzing the NBAS in the current study was different than previous studies that used the traditional seven summary cluster measures developed by Lester et al. (1982) and later revised by Jacobson et al. (1984b). These previous studies found associations between organochlorines (primarily PCBs) and increased number of abnormal reflexes, decreased autonomic maturity and habituation, and, less commonly, range of state (Jacobson et al. 1984a; Lonky et al. 1996; Rogan et al. 1986; Stewart et al. 2000).

For this analysis we hypothesized an association between PCBs and attention-associated behaviors in infancy. Previous literature suggests that prenatal PCB exposures affect attention later in childhood (Grandjean et al. 2001; Jacobson and Jacobson 1996, 2003; Peper et al. 2005; Vreugdenhil et al. 2004). A study of children born to Lake Michigan fish consumers reported associations between pre-natal PCB exposure and poorer performance on a Digit Cancellation task, which indicates difficulty with focused attention and concentration, among 11-year-old children who had not been breast-fed (Jacobson and Jacobson 2003). Associations were also reported among these same children between PCBs and poorer freedom from distractibility, a subscale of the Wechsler Intelligence Scales for Children (Jacobson and Jacobson 1996). Associations were not detected, however, between PCBs and measures of sustained attention among children in this study at 11 years of age, or at an earlier assessment made at 4 years of age (Jacobson and Jacobson 2003; Jacobson et al. 1992). The Faroe Islands study found associations between PCBs and attention measured by a continuous performance test among children 7 years of age only in the context of high mercury exposure, suggesting a potential interaction between these contaminants (Grandjean et al. 2001). A Dutch study found associations between PCBs and sustained attention among 9-year-olds, measured by a continuous performance tests (Vreugdenhil et al. 2004). Another study among adults exposed to PCBs from a contaminated building found associations with attention as well as distractibility (Peper et al. 2005).

The present analysis was designed to investigate whether an association between organochlorines and attention could be detected in early infancy. We therefore focused on *a priori* selected individual NBAS items, including supplementary items (Brazelton and Nugent 1995), as well as cluster measure outcomes that we believe reflect attention-associated skills. In addition, there is substantial overlap between childhood attention disorders [for example, the diagnosis of attention deficit hyperactivity disorder (ADHD)] and the diagnosis of motor impairment as occurs, for example, in developmental coordination disorder (Gillberg 1998; Kadesjo and Gillberg 1998). Independent of co-morbidities, children with ADHD may also have poor movement ability (Gillberg 2003; Pitcher et al. 2003), so NBAS measures of motor function were included in our analyses.

Our results show consistent inverse associations between PCB, TEQ, and DDE cord serum levels and attention-related NBAS outcomes, including alertness, quality of alert responsiveness, and cost of attention (Figure 1). The NBAS supplementary items analyzed (quality of alert responsiveness, cost of attention) were designed to capture qualitative aspects of the infant’s ability to attend to visual and auditory stimuli. For example, quality of alert responsiveness is designed to measure “processing” alertness, not just simple awake, eyes-open states. Given this specified purpose, it is notable that the association with cord serum PCBs and TEQ was particularly strong for supplementary items (Figure 1). These supplementary items were not included in the NBAS manual until 1995, well after most previous studies of PCBs and NBAS had been conducted, thereby affording an opportunity to assess a more inclusive set of attention measures in this analysis than had been possible previously.

Inverse associations were less pronounced for state- and motor-associated outcomes (Figures 2 and 4). We found little evidence for an association with the three cluster outcomes analyzed, which did not corroborate results of the previous studies conducted in Oswego, New York, and in North Carolina that found associations between PCBs and the habituation cluster (Rogan et al. 1986; Stewart et al. 2000).

The positive association observed between organochlorines and spontaneous activity is consistent with hyperactivity–impulsivity observed in experimental animal models of early-life PCB exposure (Berger et al. 2001; Holene et al. 1998; Rice 2000). Impulsive responding has been reported among humans exposed to PCBs. A study designed to dissociate response inhibition from attention found associations between PCBs and impulsive responding among children 8 and 9.5 years of age but not with sustained attention (Stewart et al. 2005). These results are consistent with earlier studies of this cohort in which an association between PCBs and response inhibition was reported at 4.5 years of age (Stewart et al. 2003). The Michigan study also found associations between PCBs and impulsivity among 11-year-olds who had not been breast-fed (Jacobson and Jacobson 2003)

We examined whether organochlorines impaired the infant’s ability to recover from the birth experience by assessing changes in performance between the birth exam and the 2-week exam. Recovery is informative of an infant’s ability to cope and adapt to the extrauterine environment, and was an originally intended application of the NBAS (Brazelton and Nugent 1995). Our findings for recovery were limited in power, however, because data were missing for either exam (both were required).

PCBs are a heterogeneous mixture of congeners, potentially with different toxicologic modes of action. A considerable strength of this study was our ability to measure up to 51 different PCB congeners. Though not presented in the results, effect sizes for the sum of 51 congeners were similar, though slightly attenuated, compared with the sum of 4 PCBs; these 4 congeners are more prevalent and likely measured with more accuracy than the sum of 51 congeners, which may explain the more attenuated effects found for the sum of 51 congeners. The correlation between the sum of 51 and the sum of 4 congeners was also quite high (0.91), and we therefore presented only the sum of the 4 congeners, which we believe adequately represents the sum of all congeners.

To take advantage of congener-specific information, we grouped congeners into structurally related classes—dioxin-like PCBs (TEQ) and non-dioxin-like PCBs (sum of 4 congeners)—that represented potentially different biologic mechanisms in their effect on neurodevelopment. That we did not find a difference in the effect of these two groups on NBAS outcomes was not surprising, because these groups were highly correlated in our study. Categorizing PCBs into such congener classes is important, however, for understanding the mode of action of these different compounds and their relative neurotoxic potency, both of which have implications for risk assessment (Schantz et al. 2003).

A barrier to understanding congener-specific effects stems from lack of knowledge of the biologic mechanism for the observed effect of PCBs on attention. Associations of PCBs with ADHD-like behaviors have been observed in rodents and nonhuman primates, however (Berger et al. 2001; Holene et al. 1998; Rice 2000). PCBs can disrupt dopaminergic functions, as reflected in alterations in dopamine levels in cell culture and in the brains of laboratory animals (Seegal et al. 1997, 2002). This is one possible mechanism whereby PCBs may affect attention-related behaviors, since decreases in cellular dopamine have been correlated with attention disorders such as ADHD (Faraone and Biederman 1998). PCBs and their hydroxylated metabolites can disrupt thyroid home-ostasis, another potential mechanism of ADHD-like effect (Hauser et al. 1998; Kimura-Kuroda et al. 2007). For example, early-life hypothyroidism is associated with subsequent ADHD-like behaviors in animal models (Negishi et al. 2005; Siesser et al. 2006). There is evidence that variations in thyroid indices or resistance to thyroid hormone may be associated with ADHD-like behavior in humans (Alvarez-Pedrerol et al. 2007; Hauser et al. 1993; Matochik et al. 1996; Stein et al. 1995), but this association has not been consistently demonstrated (Stein and Weiss 2003; Toren et al. 1997).

Serum PCB levels in the New Bedford study population were generally lower than in other birth cohort studies of PCB exposure and likely reflect more general population levels. A comparison of 10 studies of diverse locations and birth years found that the New Bedford cohort was at the low range of PCB levels (represented by PCB congener 153) relative to other populations (Longnecker et al. 2003). This would have reduced power to detect associations; that we still found consistent associations with attention indicates potential for effects at low PCB levels found in the general population.

It is unclear how much can be learned of an individual’s long-term behavior from observations in infancy. Previous studies find that infant attention is predictive of intelligence in childhood and adolescence (Sigman et al. 1986, 1997). A study of temperament in infancy (5–6 months) and toddlerhood reported significant prediction of impulsivity and inattention among 8-year-old children (Olson et al. 2002). Other studies did not find infant temperament alone to be predictive, but rather that the combination of infant temperament with parental attitude or perception was predictive of behavioral problems later in childhood (Cameron 1978; Oberklaid et al. 1993; Wasserman et al. 1990). These studies suggest that infant temperament and the interaction between the infant and his or her environment are important determinants of later behavioral outcomes.

How well the NBAS predicts attention later in childhood is also uncertain. Some studies report poor correlation between NBAS and behavioral outcomes later in childhood (Risholm-Mothander 1989; Sameroff et al. 1978), whereas another study found behavioral and reflex clusters to be good predictors of later developmental disabilities among a high-risk population (Ohgi et al. 2003). An important area for future study will be to examine whether these PCB-associated decrements in attention-related skills in infancy are transient or predict attention-related problems later in childhood.

We did not assess maternal IQ at birth, though we did measure maternal IQ with the Kaufman Brief Intelligence Test (Kaufman and Kaufman 1990) on a subset of the children that were available for testing at 8 years old. When we included maternal IQ as a covariate the exposure–outcome effect was slightly stronger, although the precision was reduced because of the smaller number of children on whom maternal IQ was available. In addition, maternal IQ was not predictive of any of the analyzed NBAS items. Final estimates are therefore reported without adjustment for maternal IQ.

This analysis was limited to subjects who were administered an NBAS exam (539 had an exam around the time of birth, 542 had an exam 1–3 weeks after birth, and 408 had both exams). This process resulted in reduced study power, particularly for the recovery analysis, which required both exams. However, we do not expect that it resulted in bias as organo-chlorine levels and covariates for these groups were very similar (data not shown).

Every effort was made to schedule infant exams in the second week of life; however, it was difficult to examine young infants at such a narrow age range in a nonclinical, population-based study. The use of a broad time window for the 2-week exam may have introduced some random error into our estimates. Exposure effect estimates for NBAS exams conducted for a more narrow window (10–17 days) were very close to estimates from the broader time window (5–22 days), suggesting that including a broader age range did not bias our results.

We evaluated a number of outcomes for this analysis as well as several categories of PCB congeners and DDE. Multiple comparisons were therefore performed and may be a limitation when interpreting positive findings. Our observation of internal consistency, however, particularly for the attention-associated outcomes, suggests that our findings were probably not attributed to chance.

In summary, we found evidence for an association between low-level prenatal organo-chlorine exposure measures and attention in early infancy. This observation is particularly notable given both the low-level PCB exposure in our study population and the limitations of behavioral assessments in young infants. The longitudinal design of the New Bedford cohort will enable us to further determine whether *a*) poor attention-associated skills in early infancy (as measured by the NBAS) persist in later childhood—that is, whether a behavioral pattern seen in infancy predicts later childhood behavioral skills; and *b*) the observed association of prenatal organochlorine exposure with attention in infancy persists in later childhood. Identifying attention-related deficits as early as infancy, and identifying potentially remediable risk factors for such deficits (for example, PCB exposure), allows for early intervention (and ultimately prevention efforts), which may be important for promoting healthy subsequent neurodevelopment.

## Figures and Tables

**Table 1:** NBAS outcome measures.

	Capacity measured	NBAS item
Individual NBAS items	Attention	Alertness
		Quality of alert responsiveness^a^
		Cost of attention^a^
	State	Consolability
		Self-quieting activity
		Hand-to-mouth facility
		Irritability
	Motor function	Elicited activity
		Spontaneous activity
		Motor maturity
NBAS clusters	Orientation	Average score for response to visual and auditory animate and inanimate stimuli, and alertness items
	Habituation	Average score for habituation to light, rattle, bell, and pin prick items
	Regulation of state	Average score for self-quieting and hand-to-mouth items
Other measures	Never in state for assessment of orientation items^b^ Recovery from birth experience^c^	

a Supplementary NBAS items.

b Child never reached alert or awake state in which he or she was able to focus attention on stimuli. Dichotomized variable as all orientation items missing, and data on at least one orientation item.

c Improved performance from birth exam to 2-week exam on individual NBAS items.

**Table 2:** Distribution of baseline characteristics for mothers and term infants with an NBAS exam approximately 2 weeks after birth, New Bedford, 1993–1998 (*n* = 542).

	No. (%)	Mean ± SD	Range
Maternal characteristics			
Age (years)	542	26.3 ± 5.5	17–40
Age category (years)			
< 20	70 (12.9)		
20–29	311 (57.4)		
30–34	117 (21.6)		
≥ 35	44 (8.1)		
Race/ethnicity (9 missing)			
White	419 (78.6)		
Black	26 (4.9)		
Hispanic	36 (6.8)		
Other	52 (9.8)		
Education (9 missing)			
≤ 11th grade	100 (18.8)		
High school graduate	204 (38.3)		
Some college	229 (43.0)		
Annual household income (38 missing)			
< $20,000	192 (38.1)		
$20–39,999	157 (31.2)		
≥ $40,000	155 (30.8)		
Marital status (9 missing)			
Married	306 (57.4)		
Never married/separated/divorced	227 (42.6)		
Parity			
None	205 (37.8)		
One	220 (40.6)		
Two or more	117 (21.6)		
Consumed local fish (9 missing)			
Yes	53 (9.9)		
No	480 (90.1)		
Smoked during pregnancy (4 missing)			
Yes	172 (32.0)		
No	366 (68.0)		
No. of cigarettes per day		2.8 ± 5.9	0–40
Alcohol consumption during pregnancy (9 missing)			
< 1 serving/month	481 (90.2)		
1–2 servings/month	14 (2.6)		
> 2 servings/month	38 (7.1)		
No. of servings per month		0.6 ± 2.8	0–38.4
Used illicit drugs before birth (12 missing)			
Yes	74 (14.0)		
No	456 (86.0)		
Born in U.S. (11 missing)			
Yes	422 (79.5)		
No	109 (20.5)		
Infant characteristics			
Gestational age (weeks)	542	39.8 ± 1.1	37–42.5
Birth weight (g)	542	3,401 ± 434	1,901–5,221
Sex			
Male	274 (50.6)		
Female	268 (49.5)		
Birth year			
1993–1994	162 (29.9)		
1995–1996	210 (38.8)		
1997–1998	170 (31.4)		
Breast-fed > 1 month (29 missing)			
Yes	191 (37.2)		
No	322 (62.8)		
Cord blood measures			
∑PCB_4_^a^ (ng/g serum)	542	0.25 ± 0.28	0.01–4.41
TEQ^b^ (pg/g lipid)	542	6.75 ± 9.73	0–151.49
DDE (ng/g serum)	542	0.48 ± 0.85	0–10.27
Pb (μ) (15 missing)	527	1.45 ± 0.97	0–9.39

a Sum of PCB congeners 118, 138, 153, 180.

b TEF-weighted sum of mono-*ortho* PCB congeners 105, 118, 156, 167, and 189.
